# Giant Ulcerated Fibroepithelial Stromal Polyp of the Vulva: A Case Report

**DOI:** 10.7759/cureus.40017

**Published:** 2023-06-05

**Authors:** Ioannis Korkontzelos, George Mpourazanis, Fatma Goshi, Romanos Vogiatzis, Daphne J Theodorou, Pantelina-Danai Korkontzelou, Eufemia Balassi, Vasiliki E Georgakopoulou, Theodora Papamitsou

**Affiliations:** 1 Department of Obstetrics and Gynecology, Ioannina State General Hospital G. Chatzikosta, Ioannina, GRC; 2 Department of Dermatology, Ernst-Moritz-Arndt University, Greifswald, DEU; 3 Department of Radiology, Ioannina State General Hospital G. Chatzikosta, Ioannina, GRC; 4 Department of Medicine, University of Sofia, Sofia, BGR; 5 Department of Pathology, Ioannina State General Hospital G. Chatzikosta, Ioannina, GRC; 6 Department of Pulmonology, Laiko General Hospital, Athens, GRC; 7 Department of Pulmonology, Sismanoglio Hospital, Athens, GRC; 8 Department of Histology-Embryology, School of Medicine, Faculty of Health Sciences, Aristotle University of Thessaloniki, Thessaloniki, GRC

**Keywords:** oncological gynecology, benign mesenchymal tumors, excision, fibroepithelial stromal polyp, vulva

## Abstract

Fibroepithelial stromal polyps (FEPs) are benign skin tumors or lesions of mesenchymal and ectodermal origin, also referred to as acrochordons. Herein, we report the case of a 45-year-old woman with a large ulcerated fibroepithelial stromal polyp extending from the right labium of the vulva. No known predisposing factor was recorded to justify the presence and rapid growth of the polyp. Antibiotic treatment was given due to inflammation, and magnetic resonance imaging was useful in establishing a diagnosis. A wide surgical excision was performed, and a histopathological examination confirmed the initial diagnosis, revealing no nuclear atypia or mitoses. The patient recovered well, and follow-up after one year showed no evidence of complications or recurrence.

## Introduction

According to the scientific literature, fibroepithelial stromal polyps (FEPs) are benign skin tumors of mesenchymal and ectodermal origin, also referred to as mesodermal stromal polyps, cellular pseudosarcomatous fibroepithelial polyps, pseudosarcoma botryoids, or acrochordons [1-3]. According to their size, FEPs are classified into skin tags if they are millimeters in diameter, fibroepithelial polyps if they are bigger (usually less than 5 cm), and giant fibroepithelial polyps if their size is larger than 5 cm [4].

They occur in approximately 25% of the population, usually as solitary lesions and less frequently as multiple lesions, and their frequency increases with age. FEPs are more commonly observed in women of reproductive age and occur most frequently in the vagina, vulva, and cervix, with only a few cases occurring in the extragenital area [3].

The pathogenesis is not yet well known, but a hormonal stimulation or chronic inflammatory process has been proposed. They are hormone-sensitive tumors, with estrogen and progesterone receptors present in the polyp’s stromal cells [2,3,5]. Histologically, they exhibit a cellular stromal component with a central vascular core and an overlying squamous epithelium. Hypocellular areas appear edematous and myxoid, containing scattered bland spindle cells, and the hypercellular component contains multinucleate or stellate cells. These cells are immunoreactive to estrogen and progesterone receptors, desmin, and occasionally smooth muscle actin [6].

Herein, we report a rare case of a 45-year-old woman with a large ulcerated fibroepithelial stromal polyp extending from the right labium of the vulva. Magnetic resonance imaging (MRI) was helpful in the definitive diagnosis, and wide excision was the treatment of choice. Considering the normal values of sex hormones, it is a rare case of a tumor developed with no certain risk factor being identified.

## Case presentation

A 45-year-old woman presented to the outpatient gynecology unit complaining of vaginal spotting and discomfort. A detailed history revealed that she had never had sexual activity and had never undergone a gynecologic examination, or Papanicolaou smear. Her menstrual history was unremarkable, and no hormonal therapy was ever administered. Her body mass index (BMI) was 24 kg/m^2^. The patient was a non-smoker and denied alcohol use. Her routine medications were bromazepam (3 mg once daily), olanzapine (5 mg once daily), venlafaxine (75 mg once daily), and aripiprazole (30 mg once daily) due to bipolar disorder.

Gynecological examination revealed a skin-colored, pedunculated, non-tender soft mass of approximately 15 cm extending from the right labium majora with inflammation, ulceration, and leukorrhea. The patient mentioned that this mass first appeared two years ago, with significant growth in the last six months. Transvaginal ultrasound revealed a normal uterus and annexes, no polycystic ovary syndrome (PCOS), and no fluid in the Douglas pouch. Body temperature was 37.2°C, and laboratory tests showed leukocytosis (leukocytes: 13.54 k/μL [reference range: 4.5-11 k/μL, neutrophils: 85%]) and a C-reactive protein of 4.53 mg/dl (reference range: 0-0.80 mg/dl) attributed to the present inflammation. Regarding the levels of sex hormones, they were all within the normal range (luteal phase of the menstrual cycle, estradiol: 116 pg/mL [normal range: 21-312 pg/mL], progesterone: 4.7 ng/mL [normal range: 1.2-15.9 ng/mL], luteinizing hormone/LH: 5.3 mIU/mL [normal range: 0.56-14 mIU/mL], follicle-stimulating hormone/FSH: 2.05 mIU/mL [normal range: 1.38-5.47 mIU/mL], testosterone: 0.29 ng/mL [normal range: 0.07-0.79 ng/mL], and prolactin: 7.44 ng/mL [normal range: 5.18-26.53 ng/mL]). Cancer biomarkers were all within the normal range (cancer antigen 125 [CA 125]: 12.3 U/mL [reference range: <35 U/mL], CA 15-3: 18 U/mL [reference range: <31.3 U/mL], CA 19-9: 22.4 U/mL [reference range: <37 U/mL], and carcinoembryonic antigen [CEA]: 2 ng/mL [reference range: <5 ng/mL]). No enlarged pelvic lymph nodes were noted from the clinical examination.

**Figure 1:**
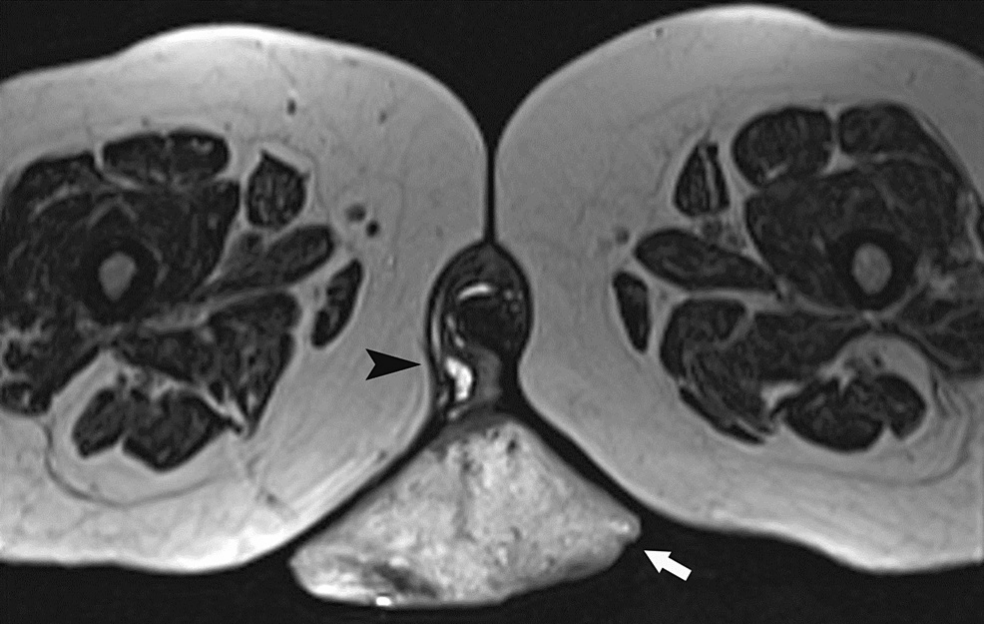
An axial T2-weighted magnetic resonance image shows a large pedunculated mass (arrow) with a stalk (arrowhead) arising from the labia majora on the right

After admission, cefoxitin was administered, and pelvic computed tomography (CT) and MRI were performed. The CT imaging revealed peripheral enhancement of the mass and the fibrovascular connection, and the MRI (with intravenous gadolinium) revealed a large exophytic mass, 10 cm x 12 cm, in contact with the lateral wall of the urethra and protruding from the labia majora on the right with intense but not homogeneous contrast enhancement. There were no abnormal lymph nodes or any free fluid in the pelvis (Figure 1). In addition, the subcutaneous fat at the contact site was intact without invasion. Pelvic organs such as the urethra were normal. These MRI findings were indicative of a benign mass [3].

**Figure 2:**
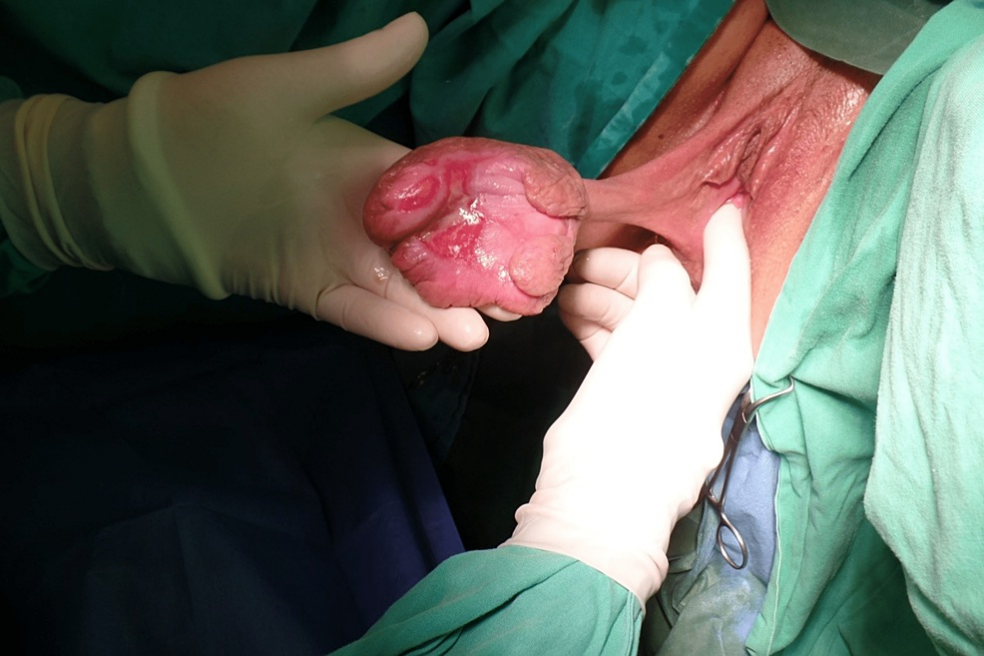
Macroscopic appearance of the giant ulcerated fibroepithelial polyp

**Figure 3:**
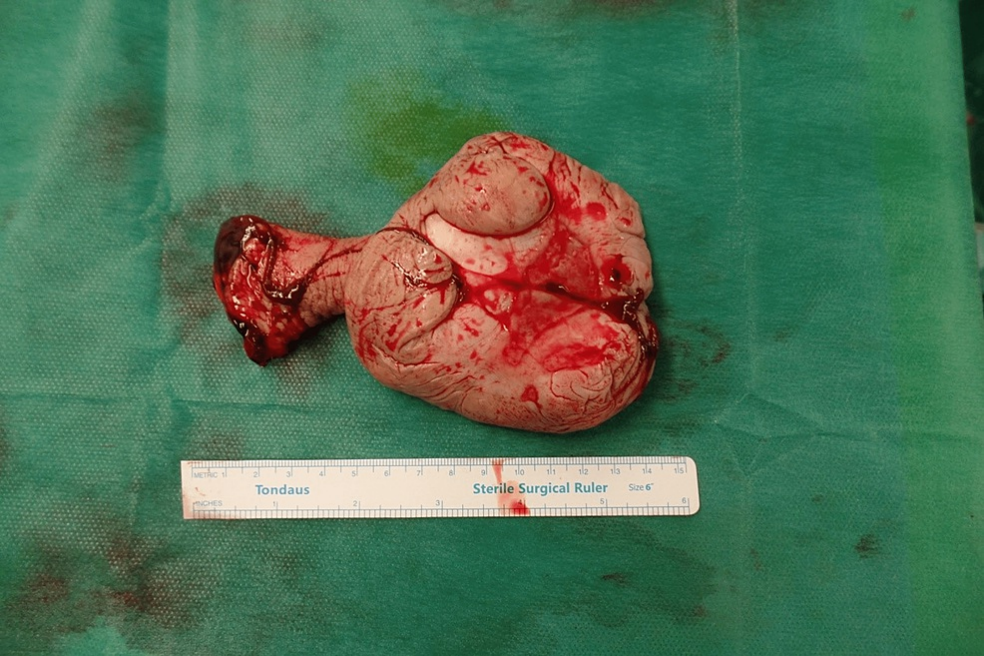
Macroscopic appearance of the excised tumor (dimensions: 10 cm x 13 cm)

Under general anesthesia, the patient was placed in the lithotomy position, and total labial excision of the ulcerated mass was performed (Figures 2, 3).

**Figure 4:**
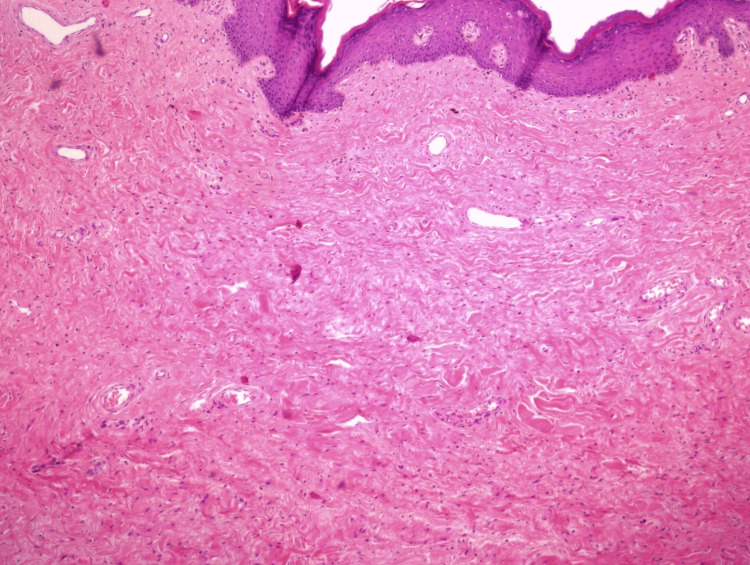
Fibroepithelial stromal polyp. Spindle cells are set within a loose collagenous myxoid-like stroma. Overlying squamous epithelium displays reactive changes.

The specimen had an interspersed polypoid formation with dimensions of 13 cm x 10 cm x 5 cm and a peduncle length of 6 cm, surrounded by centrally ulcerated skin. In cross-section, the formation showed a whitish color and a fibroelastic texture. Microscopically, the histopathology showed the morphological characteristics of a fibroepithelial stromal vulvar polyp. Its layer was made up of spindly cells and ranged from slightly cellular to subcellular, with extensive edema. No nuclear atypia, pleomorphism, or mitoses were observed. The epidermis showed central ulceration, possibly due to mechanical friction, and a moderate degree of hyperkeratosis and a slight degree of hyperacanthosis in adjacent places. Alcian Blue/periodic acid Schiff (PAS) or PAS-diastase histochemical stains revealed no mucopolysaccharide deposits. Immunohistochemically, the neoplastic cells were positive for vimentin and factor XIIIa and negative for desmin, smooth muscle actin (SMA), actin HHF-35, CD-34, CD-31, protein S100, glial fibrillary acidic protein (GFAP), neurofilament, Melan-A (Mart-1), Human Melanoma Black-45 (HMB-45), and broad-spectrum keratin AE1/AE3 (Figure 4). There was no evidence of recurrence after almost one year of follow-up by gynecologic examination.

## Discussion

Fibroepithelial stromal polyps are benign mesenchymal tumors that are usually found in the neck, axilla, and submandibular or inguinal areas but can also be found in the cervix, vagina, and vulva, and usually do not exceed the size of 5 mm [2].

Ostör et al. [7] described the first cases of vulvar fibroepithelial polyps. More specifically, Ostör et al. reported eight FEPs arising from the vulva, underlining the importance of their differentiation from sarcoma botryoides, which they resemble both macroscopically and microscopically. In this case series, two cases recurred after incomplete excision [7].

Lozano-Peña et al. discussed the case of a 23-year-old woman with a nine-year history of a gradually increasing vulvar tumor that was surgically excised, and histopathology revealed the diagnosis of an FEP [4]. Kurniadi et al. reported a case of a 28-year-old woman with multiple giant FEPs. More specifically, they found two FEPs with dimensions of 20 cm x 12 cm x 8 cm and 9 cm x 4 cm x 2 cm located on both sides of her vulva [2]. In this case, the first presentation was an itchy swelling on her left vulva [2].

Ogura et al. [8]. reported a case of a 23-year-old woman with irregular bleeding and a mass on the vulva apparently unrelated to intra-abdominal organs and urogynecology. After performing an excision accompanied by a laparoscopic myomectomy, histopathology demonstrated that the lesion on the vulva was a 16 cm ×11 cm × 6 cm FEP weighing 700 g.

There are many other vulvovaginal soft tissues or lesions that can share similar features. The diagnostic algorithm involves physical examination, histopathology examination, and imaging such as a CT or MRI scan in order to delineate the origin and extent of the lesion by evaluating its blood supply and flow. The differential diagnosis includes aggressive angiomyxoma, angiomyofibroblastoma, sarcoma, superficial cervicovaginal myofibroblastoma, cellular angiofibroma, perineurinoma, botryoid embryonal rhabdomyosarcoma, and squamous cell carcinoma [2,3]. These masses show low signals on the T1-weighted (T1W) image and high signals on the T2-weighted MRI (T2W MRI) [9]. Although the MRI findings of FEPs of the vulva are often similar to those of the aforementioned masses, an FEP should be considered when radiological images show the following features: stratiform hypointense areas surrounded by patchy hyperintense areas on T2W MRI and hyperintense areas on T1W MRI [10].

The microscopic examination of the polyp is of critical importance in excluding malignancies. Histologically, the most characteristic features of this type of polyp are the presence of stellate cells and multinucleate stromal cells at the epithelium and stromal interface [6,11].

The ideal method for treating large polyps is complete surgical excision. However, incomplete resection may lead to disease recurrence, so long-term follow-up is important. Small-sized polyps can be treated with cryotherapy or cauterization [8].

In our case, the histopathology reported no malignancy, and there was no evidence of recurrence after almost one year of follow-up. However, some questions remain unanswered. In our patient, no certain predisposing factors were present. The woman had normal menstruation, was never pregnant, was not obese, and did not suffer from diabetes. Moreover, although the patient was receiving drugs that may lead to some changes in hormone concentrations, which in turn may lead to tumor development, the levels of hormones were all within the normal range [12-14]. On ultrasound, the ovaries appeared normal with no signs of PCOS, and she had never been on hormonal therapy. Furthermore, it still has to be explained why, in certain cases, rapid polyp growth is attributed to hormones (adolescence), while in other cases, the mass grows steadily for a period of time and suddenly its growth increases rapidly without any obvious cause. Future research on these tumors is needed to shed light on their pathogenetic mechanisms.

It is worth mentioning that such a tumor has a psychological impact on affected women. Studies have shown that the presence of a mass with the suspicion of gynecological cancer in a woman’s medical history is related to a high risk of developing depression, anxiety, and adjustment disorders [15].

## Conclusions

This is a rare case of fibroepithelial stromal polyps of an unclear origin. Future cases may provide additional information on the pathogenesis and growth of these superficial stromal proliferation polyps. Prompt treatment and definitive diagnosis are important to manage these stromal polyps and, of course, the patient’s anxiety.

## References

[1] Schoolmeester JK, Fritchie KJ (2015). Genital soft tissue tumors. J Cutan Pathol.

[2] Kurniadi A, Rinaldi A, Yulianti H, Bazar AR, Prasetyawati RD, Tjandraprawira KD (2022). Multiple vulvar giant fibroepithelial polyps: a rare case occurrence. Case Rep Obstet Gynecol.

[3] Yoo J, Je BK, Yeom SK, Park YS, Min KJ, Lee JH (2019). Giant fibroepithelial stromal polyp of the vulva: diffusion-weighted and conventional magnetic resonance imaging features and pathologic correlation. J Pediatr Adolesc Gynecol.

[4] Lozano-Peña AK, Lamadrid-Zertuche AC, Ocampo-Candiani J (2019). Giant fibroepithelial polyp of the vulva. Australas J Dermatol.

[5] Sari R, Akman A, Alpsoy E, Balci MK (2010). The metabolic profile in patients with skin tags. Clin Exp Med.

[6] Pharaon M, Warrick J, Lynch MC (2018). Fibroepithelial stromal polyp of the vulva: case report and review of potential histologic mimickers. Int J Gynecol Pathol.

[7] Ostör AG, Fortune DW, Riley CB (1988). Fibroepithelial polyps with atypical stromal cells (pseudosarcoma botryoides) of vulva and vagina. A report of 13 cases. Int J Gynecol Pathol.

[8] Ogura N, Inagaki M, Yasuda R, Yoshida S, Maeda T (2022). A vaginal fibroepithelial stromal polyp: a case report with magnetic resonance images. BJR Case Rep.

[9] Amin A, Amin Z, Al Farsi AR (2018). Septic presentation of a giant fibroepithelial polyp of the vulva. BMJ Case Rep.

[10] Kato H, Kanematsu M, Sato E, Ito N, Furui T, Hirose Y (2010). Magnetic resonance imaging findings of fibroepithelial polyp of the vulva: radiological-pathological correlation. Jpn J Radiol.

[11] Kurniawati EM, Djunaidi F, Kurniasari N (2022). Giant fibroepithelial polyps of the vulva in a woman with uterine myoma and primary infertility: a case report and literature review. Am J Case Rep.

[12] D'Armiento M, Bisignani G, Reda G (1981). Effect of bromazepam on growth hormone and prolactin secretion in normal subjects. Horm Res.

[13] Albaugh VL, Henry CR, Bello NT, Hajnal A, Lynch SL, Halle B, Lynch CJ (2006). Hormonal and metabolic effects of olanzapine and clozapine related to body weight in rodents. Obesity (Silver Spring).

[14] Kelly DL, Powell MM, Wehring HJ (2018). Adjunct aripiprazole reduces prolactin and prolactin-related adverse effects in premenopausal women with psychosis: Results from the DAAMSEL clinical trial. J Clin Psychopharmacol.

[15] Kostev K, Jacob L, Kalder M (2017). Risk of depression, anxiety, and adjustment disorders in women with a suspected but unconfirmed diagnosis of breast or genital organ cancer in Germany. Cancer Causes Control.

